# Supplementary figures and images for: High expression of SLC26A6 in the kidney may contribute to renal calcification via an SLC26A6-dependent mechanism
Source: PeerJ. 2018 Jul 3;6:e5192. doi: 10.7717/peerj.5192 (PMC6034601; doi:10.7717/peerj.5192)

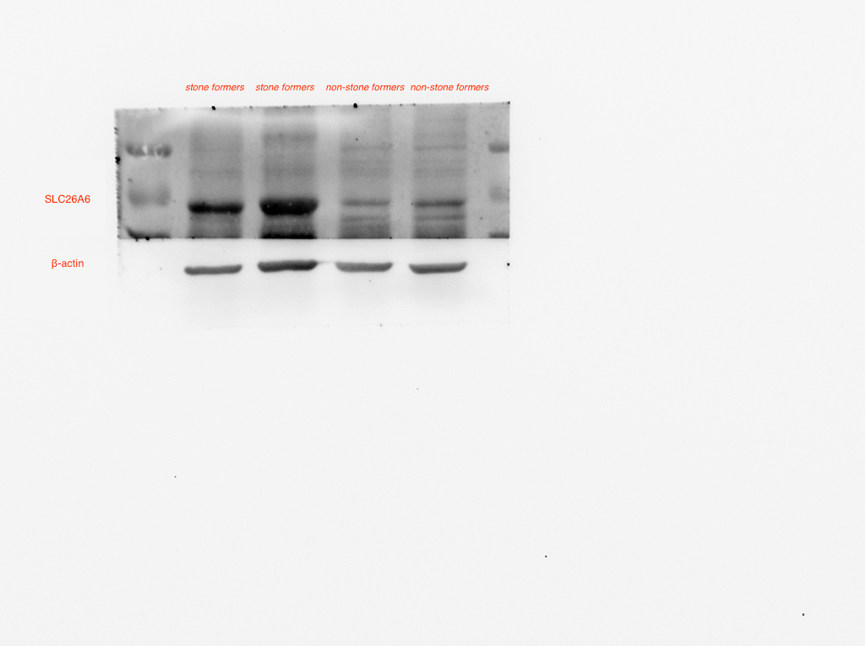

Supplement: Supplemental Information 2 [file peerj-06-5192-s002.zip › Fig1B.png]

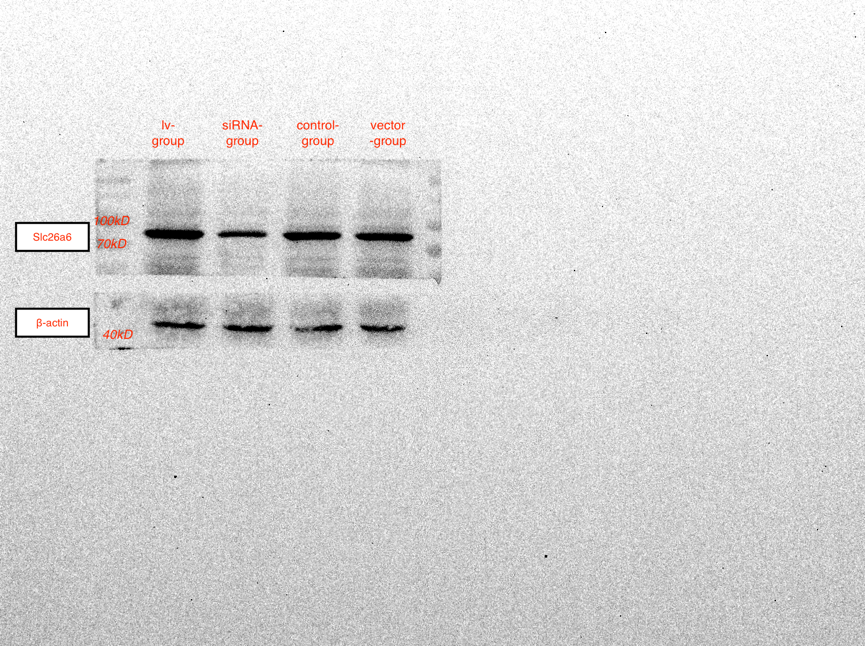

Supplement: Supplemental Information 2 [file peerj-06-5192-s002.zip › Fig3B.png]

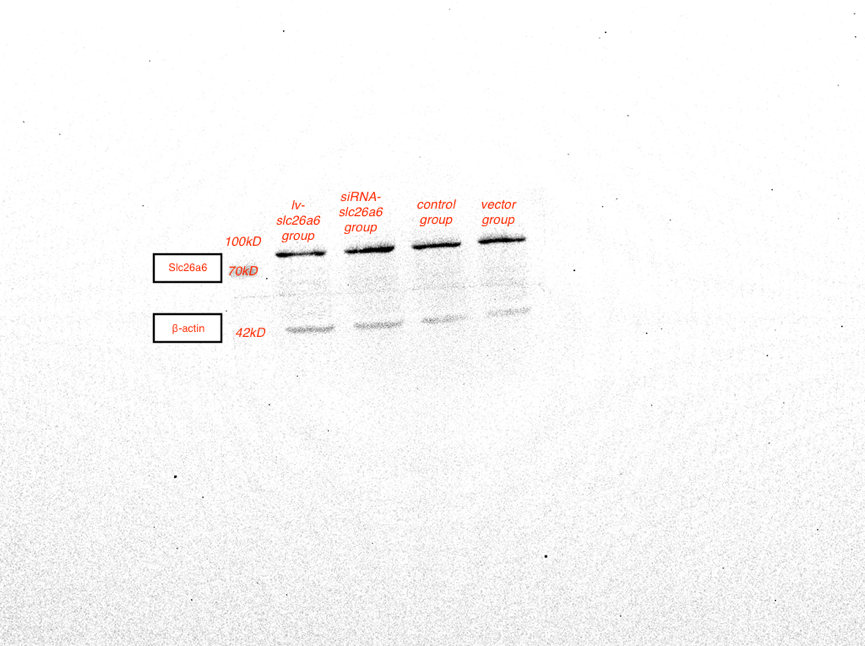

Supplement: Supplemental Information 2 [file peerj-06-5192-s002.zip › Fig3A.png]

S1

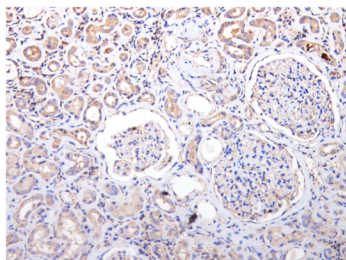

S2

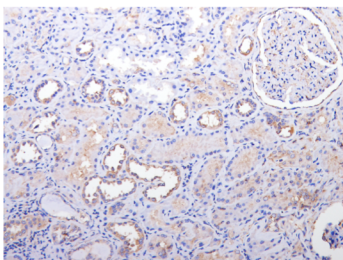

S3

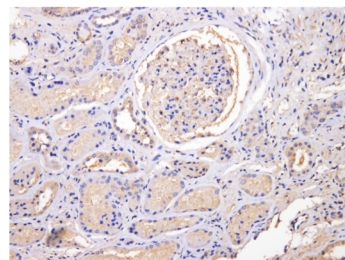

S4

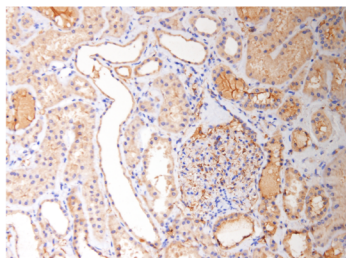

S5

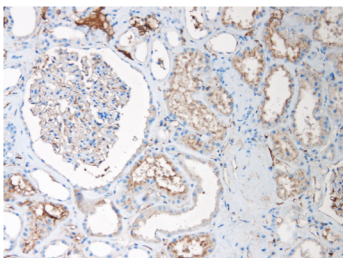

S6

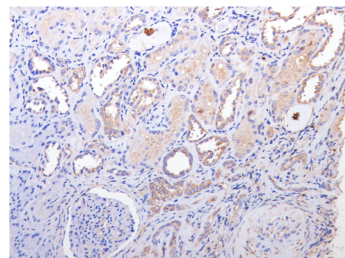

S7

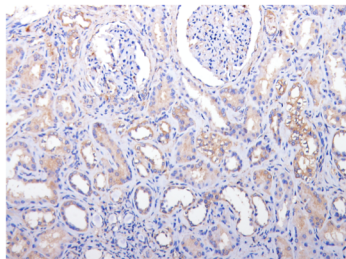

S8

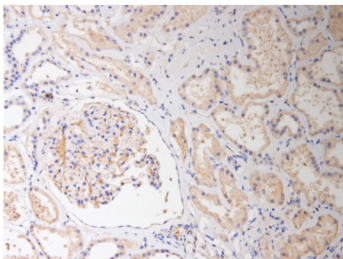

S9

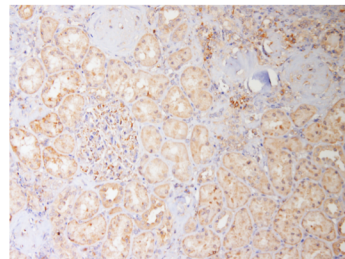

S10

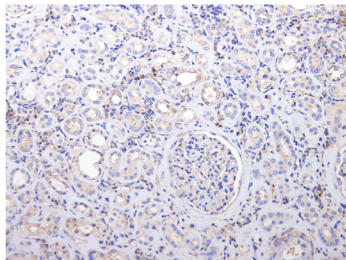

Supplement: Supplemental Information 3 — Kidney tissues from stone formers were stained by IHC assay to detected SLC26A6 expression. The brown part represents the expression of SLC26A6 (magnification: X200). [file peerj-06-5192-s003.pdf]

C1

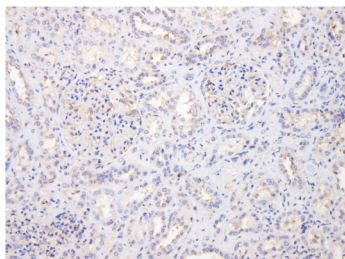

C2

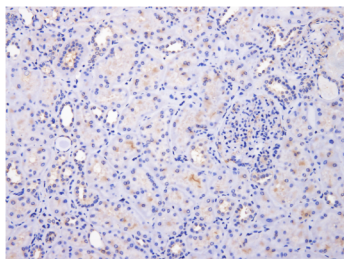

C3

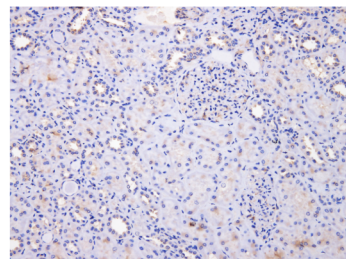

C4

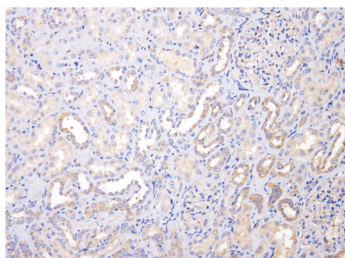

C5

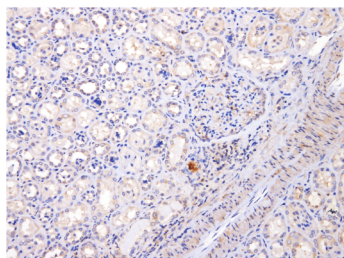

C6

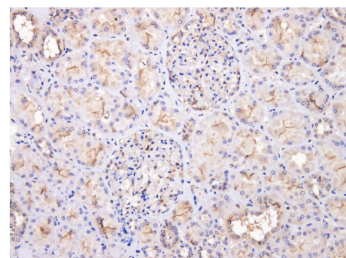

C7

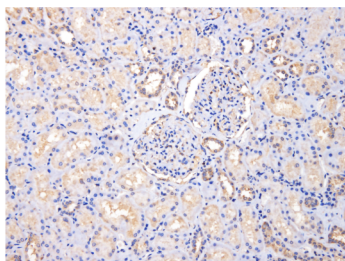

C8

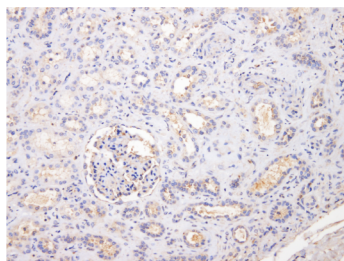

C9

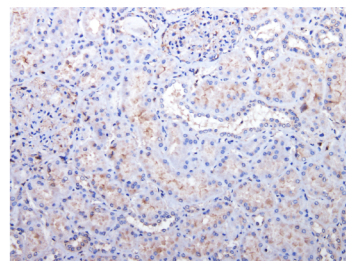

C10

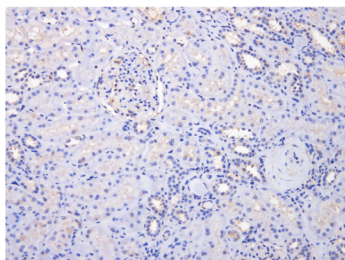

Supplement: Supplemental Information 4 — Kidney tissues from non-stone formers were stained by IHC assay to detected SLC26A6 expression. The brown part represents the expression of SLC26A6 (magnification: X200). Compare to the Fig. S2, mean pixel of SLC26A6 was lower in non-stone former groups. [file peerj-06-5192-s004.pdf]
